# Epigenetic biomarkers of cold and heat syndromes of rheumatoid arthritis: Combining DNA hydroxymethylation and mRNA‐sequencing

**DOI:** 10.1111/1756-185X.14480

**Published:** 2022-11-02

**Authors:** Nian Liu, Weitian Yan, Xianna Yang, Ling Zhang, Shaoqin Chen, Jing Zhang, Xingqiang Wang, Jiangyun Peng

**Affiliations:** ^1^ The First Clinical Medical College of Yunnan University of Chinese Medicine Kunming China; ^2^ The First Affiliated Hospital of Yunnan University of Chinese Medicine Kunming China

Syndrome (*Zheng* in Mandarin Chinese) is the induction of disease attributes in the theory of traditional Chinese medicine (TCM). This is often used to explain why patients with the same disease show different symptoms. Cold and heat represent the two basic tendencies of a syndrome; therefore, the identification of cold and heat syndromes is key to the diagnosis and treatment of rheumatoid arthritis (RA) according to TCM. However, a traditional thinking model of cold and heat syndromes identifies a reliance on the clinical manifestations, which is inevitably affected by the subjectivity of doctors. It is important to identify objective biomarkers to improve accuracy in the identification of cold and heat syndromes of RA. Previous studies have identified differences in gene expression and function between cold and heat syndromes of RA, suggesting that different syndromes possess a unique biological basis.^1^, ^2^ However, the biological basis is large and complex; therefor constructing a “disease–syndrome–biomaker” network using modern technology to preserve the characteristics of TCM and also improve the objectivity and applicability in syndrome identification is challenging

DNA hydroxymethylation is an epigenetic marker that has attracted increasing attention recently. As a bridge between methylation and demethylation, DNA hydroxymethylation drives demethylation, indicating the reactivation of gene transcriptional expression.^3^ More importantly, DNA hydroxymethylation is sensitive to environmental stimuli, the state of DNA hydroxymethylation modification is able to change under different environmental stimuli, resulting in phenotypic differences.^4^ This regulatory mechanism is consistent with the concept of TCM, whereby “environmental differences can break the balance between cold and heat, causing differences in external clinical symptoms in patients with RA”, inferring that it is feasible to explore the epigenetic biomarkers of cold and heat syndromes of RA from DNA hydroxymethylation and gene transcription. The aim of our study was to identify epigenetic biomarkers of cold and heat syndromes of RA by combining DNA hydroxymethylation sequencing and transcriptome sequencing. Our study was approved through ethical review by the First Affiliated Hospital of Yunnan University of Traditional Chinese Medicine (ethical batch number: the scientific research [2020] Ethical Review [005]‐02).

The study was conducted as follows. Ten patients with RA cold syndrome and 10 with heat syndrome were recruited according to the *Guideline for Diagnosis and Treatment of Rheumatoid Arthritis Based on TCM Syndromes*.^5^ We also included 10 healthy participants. The general situation of these groups is shown in Table 1. Next, 10 mL peripheral venous blood was extracted from each participant to isolate peripheral blood mononuclear cells; DNA and RNA were further extracted. Hydroxymethylated DNA fragments were obtained through DNA hydroxymethylation immunoprecipitation (hMeDIP), and mRNA was obtained through enzyme digestion. The Illumina high‐throughput sequencing platform was used to generate the library, in addition to hMeDIP sequencing (hMeDIP‐seq) and mRNA sequencing (mRNA‐seq). Bioinformatics analysis was used to compare the changes in DNA hydroxymethylation and transcriptional expression between cold and heat syndrome genes. Remarkably altered genes at DNA hydroxymethylation were used in Gene Ontology and the Kyoto Encyclopedia of Genes and Genomes pathway enrichment. Genes significantly altered at both the DNA hydroxymethylation and transcription expression levels were identified as epigenetic biomarkers of cold and heat syndromes of RA and the correlation was preliminarily analyzed.

**TABLE 1:** General information of participants

Category	Healthy	Cold	Heat
Age (y), mean ± SD	45.10 ± 11.41	44.10 ± 13.11	46.70 ± 9.99
Gender, *n*			
Male	2	2	2
Female	8	8	8
Nationality, *n*			
Han ethnicity	9	10	9
Other ethnic groups	1	10	1
Course of RA (mo), mean ± SD		9.80 ± 7.16	10.90 ± 8.50
DAS28 score, mean ± SD		3.37 ± 0.21	3.87 ± 0.29
Classification of joint function, *n*			
Level I		8	7
Level II		2	3
Level III		0	0
X‐ray staging of wrists, *n*			
Phase I		7	7
Phase II		2	3
Phase III		1	0
TCM syndrome classification, *n*			
Mild		3	3
Moderate		2	4
Severe		5	3

*Note*: Healthy indicates healthy participants. Cold indicates patients with rheumatoid arthritis (RA) cold syndrome. Heat indicates patients with RA cold syndrome.

Results demonstrated that genomes with significant hydroxymethylation alteration tended to have a higher hydroxymethylation level (Figure S1A). Among 754 differential genes screened, 737 genes showed upregulation (logFC ≥ 1, *P* < 10^−4^), involved in 32 pathways including the Wnt signaling pathway, T helper type 17 cell differentiation pathway, and neuroactive ligand‐receptor interaction (Figure S1B). The expression of 17 genes was downregulated (logFC ≤ −1, *P* < 10^−4^) and related to African trypanosomiasis, Malaria, and Axonal guidance pathways (Figure S1C). There were 125 genes with significant hydroxymethylated changes from patients with heat syndrome (Figure S1D). Among which, 64 genes indicated upregulated expression (logFC ≥ 1, *P* < 10^−4^) and enriched Arginine and proline metabolism (Figure S1E). The remaining 61 genes with downregulated expression (logFC ≤ −1, *P* < 10^−4^) were enriched in 8 signaling pathways, including Neuroactive ligand‐receptor interaction and Ovarian steroidogenesis (Figure S1F). It is worth noting that the Neuroactive receptor‐ligand interaction metabolic pathway was regulated by the upregulated gene in cold syndrome. Hence, upregulation or downregulation on the DNA hydroxymethylation level of the Neuroactive receptor‐ligand interaction metabolic pathway may be an important regulatory method for conversion between cold and heat syndromes of RA.

Quantitative analysis of transcriptional expression indicated that among the cold syndrome, heat syndrome, and healthy participants, the largest number of average transcriptional expression quantification (FPKM) occurred in cold syndrome genes, especially compared with that in heat syndrome ones (*P* < .05, *P* < .001, Figure S2A) at both gene and transcript levels. This correlates with overall upregulation of DNA hydroxymethylation and activation of gene transcription expression in cold syndrome.

Associated with the hMeDIP‐seq and mRNA‐seq, a total of 17 signature genes of cold syndrome that were remarkably altered in DNA hydroxymethylation and transcriptional expression were obtained, covering *NKRF*, *ACIN1*, *RAPGEF1*, *DNMT3A*, *CXCR3*, *WDR6*, *PRKD2*, *TGFBR1*, *DNAJB11*, *CIZ1*, *ZNF641*, *MIOS*, *CHD3*, *FKBP5*, *CIRBP*, *ICE1*, and *PLEC*. Among these, *CIRBP* encoded cold‐inducible RNA‐binding protein (CIRBP), which expression was upregulated in cold environments or after cold stimulation.^6^ Patients with cold syndrome often indicate that they receive cold environment stimulation for long periods of time, and a cold joint feeling is the most typical symptom. Our study suggests that the *CIRBP* of cold syndrome is highly hydroxymethylated (logFC = 1.15, *P* ≤.05, FPKM ≥ 0.05), and transcriptional expression is significantly upregulated (logFC = 1.50, *P* ≤.05, FPKM ≥ 0.05). Hence, *CIRBP* may be the most representative epigenetic biomarker gene of cold syndrome of RA.

*TIRAP*, *SMOX*, *MSRB1*, *LAIR1*, and *GAS7* were selected as the 5 signature genes of heat syndrome of RA. *TIRAP* encodes the Toll/interleukin‐1 receptor domain‐containing adapter protein (TIRAP), which activates Toll‐like (TLR) ‐dependent inflammatory signaling either MyD88‐dependent or ‐independent, activates NF‐κB and MAPK, induces dendritic cell maturation, and triggers downstream inflammatory cascade.^7^ Arthritis with heat syndrome exhibited more severe inflammation and highly expressed TLRs/MyD88 than other syndromes.^8^ Given its high hydroxymethylation (logFC = 1.07, *P* ≤ .05, FPKM ≥ 0.05) and upregulated transcriptional expression (logFC = 0.95, *P* ≤ .05, FPKM ≥ 0.05) of *TIRAP*, *TIRAP* may be the most important epigenetic biomarker gene for targeted identification of RA heat syndrome.

Correlation analysis demonstrated both positive and negative correlation between DNA hydroxymethylation and transcriptional expression (Figure S2B,C) for signature genes. This suggests that a previous conclusion, whereby upregulation of DNA hydroxymethylation resulted in the activation of transcriptional expression, may be controversial. In addition, whether the result was influenced by the threshold of differential hydroxymethylation modification and transcript expression should also be considered.

In conclusion, our study confirmed that unique epigenetic biomarker genes of cold and heat syndromes of RA exist, and these genes, together with the encoded proteins, constitute the “disease‐syndrome‐biomarker” epigenetic regulatory network. Molecular biology experiments will be conducted to verify the effectiveness and more specific regulatory mechanisms for these epigenetic biomarkers in a future study.

## AUTHOR CONTRIBUTIONS

NL conducted the majority of the experiments and wrote the manuscript, YW recruited the participants, they contributed equally to this work and share first authorship. JP revised the manuscript. XW designed the study. XY, LZ, SC, and JZ preformed the experiment. All authors read and approved the final manuscript.

## FUNDING INFORMATION

This study was supported by the National Natural Science Foundation of China (Nos 81960863 and 82160901), and Yunnan Clinical Research Center for Rheumatism of Traditional Chinese Medicine (No. 202002AA310005).

## CONFLICT OF INTEREST

None.

## Supporting information


Figure S1

Figure S2

Figures S1–S2
