# Supplementary figures and images for: Epigenetic biomarkers of cold and heat syndromes of rheumatoid arthritis: Combining DNA hydroxymethylation and mRNA‐sequencing
Source: Int J Rheum Dis. 2022 Nov 2;26(2):393–5. doi: 10.1111/1756-185X.14480 (PMC10092574; doi:10.1111/1756-185X.14480)

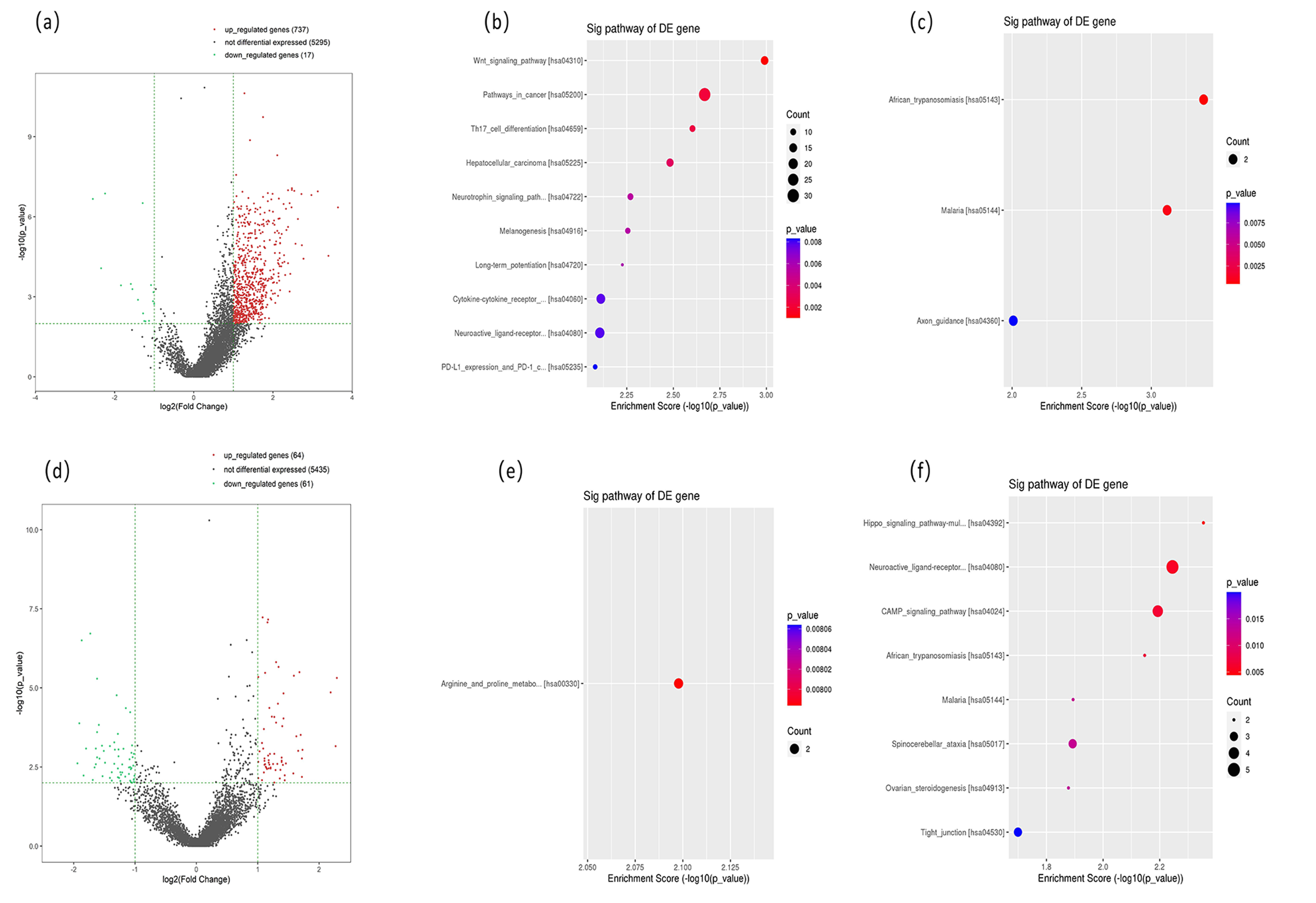

Supplement: Supplementary file 1 — Figure S1 [file APL-26-393-s001.tif]

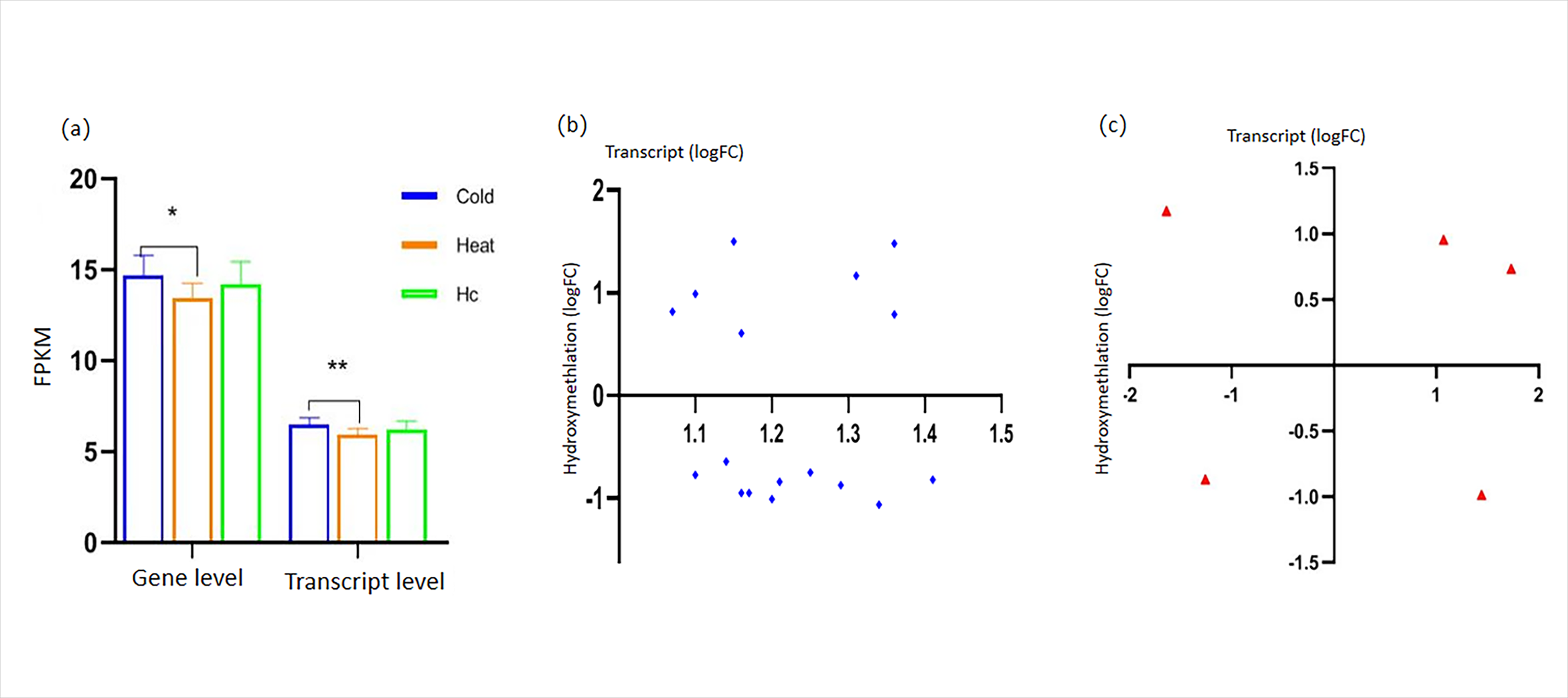

Supplement: Supplementary file 2 — Figure S2 [file APL-26-393-s002.tif]
