# Supplementary material for: Epigenetic biomarkers of cold and heat syndromes of rheumatoid arthritis: Combining DNA hydroxymethylation and mRNA‐sequencing
Source: Int J Rheum Dis. 2022 Nov 2;26(2):393–5. doi: 10.1111/1756-185X.14480 (PMC10092574; doi:10.1111/1756-185X.14480)
Supplement: Supplementary file 3 — Figures S1–S2 [file APL-26-393-s003.docx]

**FIGURE S1.** DhMRs clustering and KEGG pathway enrichment of cold and heat syndromes. (a) DhMRs clustering of cold syndrome. (b) KEGG pathway enrichment of upregulated hydroxymethylation genes of cold syndrome. (c) KEGG pathway enrichment of downregulated hydroxymethylation genes of cold syndrome. (d) DhMRs clustering of heat syndrome. (e) KEGG pathway enrichment of upregulated hydroxymethylation genes of heat syndrome. (f) KEGG pathway enrichment of downregulated hydroxymethylation genes of heat syndrome. *Note*: DhMRs indicates differentially hydroxymethylated regions. In panels (a) and (d), red dots indicates upregulated hydroxymethylation genes, and green dots indicates downregulated hydroxymethylation genes.

**FIGURE S2.** Quantitative analysis of transcriptional expression and distribution of epigenetic biomarker genes. (a) Quantification of transcriptional expression at both gene and transcript level. (b) Four quadrants distribution map of epigenetic biomarker genes of cold syndrome. (c) Four quadrants distribution map of epigenetic biomarker genes of heat syndrome. *Note:* Cold indicates cold syndrome of RA. Heat indicates heat syndrome of RA. Hc indicates healthy participants. Data are presented as the mean ± SD, **P* < .05, ***P* < .01.
